# Resveratrol analog piceatannol restores the palmitic acid-induced impairment of insulin signaling and production of endothelial nitric oxide via activation of anti-inflammatory and antioxidative heme oxygenase-1 in human endothelial cells

**DOI:** 10.3892/mmr.2015.3553

**Published:** 2015-03-26

**Authors:** SUN-OH JEONG, YONG SON, JU HWAN LEE, YONG-KWAN CHEONG, SEONG HOON PARK, HUN-TAEG CHUNG, HYUN-OCK PAE

**Affiliations:** 1 Department of Microbiology and Immunology, Iksan 570-749, Republic of Korea; 2 Department of Anesthesiology and Pain Medicine, Iksan 570-749, Republic of Korea; 3 Institute for Metabolic Disease, Wonkwang University School of Medicine, Iksan 570-749, Republic of Korea; 4 Department of Biological Science, University of Ulsan, Ulsan 680-749, Republic of Korea

**Keywords:** piceatannol, palmitic acid, endothelial dysfunction, insulin resistance, endothelial nitric oxide synthase, heme oxygenase-1

## Abstract

Growing evidence suggests that the elevation of free fatty acids, including palmitic acid (PA), are associated with inflammation and oxidative stress, which may be involved in endothelial dysfunction, characterized by the reduced bioavailability of nitric oxide (NO) synthesized from endothelial NO synthase (eNOS). Heme oxygenase-1 (HO-1) is important in the preservation of NO bioavailability. Piceatannol (Pic), with similar chemical structure to resveratrol, is suggested to possess similar protective effects as resveratrol. In the present study, human umbilical vein endothelial cells (HUVECs), stimulated with PA, were used to examine the endothelial protective effects of Pic. Pic increased the expression of HO-1 via nuclear factor erythroid-2-related factor-2 activation in the HUVECs, and decreased the PA-induced secretions of interleukin-6 and tumor necrosis factor-α, and the formation of reactive oxygen species ROS via inhibition of NF-κB activation. Notably, following inhibition of HO-1 activity by tin protoporphryin-IX, Pic did not prevent cytokine secretion, ROS formation, and NF-κB activation in the PA-stimulated HUVECs. PA attenuated insulin-mediated insulin receptor substrate-1 (IRS-1) tyrosine phosphorylation, leading to decreased glucose uptake, and phosphorylation of eNOS, leading to a reduction in the production of NO. Pic effectively mitigated the inhibitory effects of PA on the insulin-mediated phosphorylation of IRS-1 and eNOS, which was not observed following inhibition of HO-1 activity. The results of the present study suggested that Pic may have the potential to prevent PA-induced impairment of insulin signaling and eNOS function, by inducing the expression of the anti-inflammatory and antioxidant, HO-1.

## Introduction

Endothelial dysfunction, characterized by decreased bioavailability of nitric oxide (NO) and impaired endothelium-dependent vasodilation, is a primary cause of vascular complications in type 2 diabetes mellitus, and is one of the major risk factors of cardiovascular diseases (1). The elevation of circulating free fatty acids (FFAs), as observed in individuals with obese insulin resistance and type 2 diabetes mellitus, is associated with impaired endothelium-dependent vasodilation (2), suggesting a link between FFA and endothelial dysfunction. At a molecular level, FFAs activate nuclear factor-κB (NF-κB) via their ligation of Toll-like receptor 4 (TLR4), leading to release of pro-inflammatory cytokines, including interleukin (IL)-6 and tumor necrosis factor-α (TNF-α), and the formation of reactive oxygen species (ROS) (3), suggesting that the abnormal elevation of circulating FFAs may cause endothelial dysfunction by inducing inflammation and oxidative stress in vascular tissues. FFAs also prevent insulin receptor substrate-1 (IRS-1) tyrosine phosphorylation in cultured endothelial cells, which subsequently reduces the insulin-dependent activation of endothelial NO synthase (eNOS) and production of NO (4). As a major component of dietary saturated fat and 20% of the total serum FFAs (5,6), palmitic acid (PA) is often used to induce endothelial dysfunction via NF-κB- and IRS-1-dependent pathways in endothelial cell culture models (7).

Heme oxygenase-1 (HO-1), an inducible enzyme with potent antioxidant and anti-inflammatory properties, catalyzes the degradation of heme to carbon monoxide, biliverdin and free iron, with biliverdin subsequently being metabolized to bilirubin by biliverdin reductase (8). The nuclear factor E2-related factor 2 (Nrf2) is important in the transcriptional activation of HO-1 and several other genes (9). Upon activation, Nrf2 enters the nucleus, where it binds to AU-rich elements in the HO-1 promoter to trigger gene expression (9). Nrf2 has been observed to regulate the induction of the expression of HO-1 in response to certain naturally occurring compounds, including curcumin and resveratrol (10). Several studies have confirmed the protective role of HO-1 in several pathological states, including endothelial dysfunction (8,9).

Resveratrol is a naturally occurring stilbene, which is present in various types of food and beverage, and has attracted increasing attention due to its multiple beneficial properties, including anti-inflammatory and antioxidant activities (11). There are several naturally occurring stilbene-like compounds, which are structurally similar to resveratrol. Piceatannol (Pic), which was first isolated from the seeds of *Euphorbia lagascae* (Euphorbiaceae), is a naturally occurring hydroxylated analog of resveratrol and has been also identified as a resveratrol metabolite (12). Based on its structural similarity to resveratrol and formation through *in vivo* metabolism of resveratrol (11), it has been hypothesized that Pic may have biological activities similar to those of resveratrol. The only difference between Pic (3,5,4′,3′-*trans*-trihydroxystilbene) and resveratrol (3,5,4′-*trans*-trihydroxystilbene) is the presence of an additional hydroxyl group in one of the aromatic rings of Pic (11). Although Pic, partly due to this difference, has increased antioxidant activity compared with resveratrol (11,12), whether Pic exerts other biological effects similar to those of resveratrol remains to be elucidated.

It has been previously demonstrated that resveratrol prevents endothelial dysfunction in cultured human umbilical vein endothelial cells (HUVECs) exposed to high glucose (13,14) and in trauma-hemorrhaged animals (15). However, whether Pic, as with resveratrol, prevents endothelial dysfunction remains to be elucidated. The present study aimed to investigate this, using PA as one of the predominant mediators to induce endothelial dysfunction in HUVECs, by inducing inflammation and ROS formation and by reducing insulin- mediated NO bioavailability. Using this *in vitro* endothelial dysfunction model, the results demonstrated that Pic induced the Nrf2-dependent expression of HO-1, an anti-inflammatory and antioxidant, which inhibited the PA-induced inflammatory response and formation of ROS, and attenuated the PA-induced reduction in insulin-mediated eNOS activation and production of NO.

## Materials and methods

### Materials and antibodies

The 3,3′,4,5′-tetrahydroxy-*trans*-stilbene (piceatannol; Pic), hemin, 3-(4,5-Dimethylthiazol-2-yl)-2, 5-diphenyltetrazolium bromide (MTT), PA, bovine serum albumin (BSA), dimethyl sulfoxide (DMSO), and tin protoporphryin-IX (SnPP) were purchased from Sigma-Aldrich (St. Louis, MO, USA). Antibodies rabbit polyclonal Nrf2 (cat. no. sc-722; 1:500 dilution), rabbit polyclonal Lamin B (cat. no. sc-20682; 1:1,000 dilution) and mouse monoclonal β-actin (cat. no. sc-47778; 1:1,000 dilution) and Nrf2 small interfering (si)RNA (cat. no. sc-37030) and control siRNA (cat. no. sc-37007) were purchased from Santa Cruz Biotechnology, Inc. (Santa Cruz, CA, USA). Horseradish-peroxidase (HRP)-conjugated secondary antibodies against rabbit (cat. no. 7074; 1:1,000 dilution) and mouse (cat. no. 7076; 1:1,000 dilution) immunoglobulin (Ig) G and the following primary antibodies: Rabbit monoclonal phosphorylated (p)-NF-κB p65 (cat. no. 93H1; 1:1,000 dilution), rabbit monoclonal NF-κB p65 (cat. no. C22B4; 1:1,000 dilution), rabbit monoclonal p-eNOS (cat. no. C9C3; 1:1,000 dilution) and rabbit polyclonal eNOS (cat. no. 9572; 1:1,000 dilution) were obtained from Cell Signaling Technology, Inc. (Beverly, MA, USA). The following rabbit polyclonal antibodies targeting p-IRS-1 (cat. no. BS4633; 1:1,000 dilution) and IRS-1 (cat. no. E306; 1:1,000 dilution) were obtained from Bioworld Technology (St. Louis Park, MN, USA). The PA was dissolved in absolute ethanol at 200 mM as a stock solution and was further diluted with medium containing 10% BSA, to obtain a concentration of 5 mM, prior to use. All other reagents, unless otherwise stated, were purchased from Sigma-Aldrich.

### Cell culture

All of the investigations performed in the present study were approved by the Research Ethics Committee of Wokwang University (Iksan, South Korea). The HUVECs were purchased from Cascade Biologics Inc. (Portland, OR, USA). The HUVECs were grown in RPMI-1640 medium (Sigma-Aldrich), supplemented with 10% fetal bovine serum, streptomycin (100 U/*μ*l) and penicillin (100 U/*μ*l) in an atmosphere of 5% CO_2_ and 95% humidified air at 37°C. The medium was renewed every 2 days until the cells had grown to confluence. The confluent cells were detached using trypsin-EDTA (0.05% trypsin, 0.02% EDTA), and cells between passages three and seven were used in the subsequent experiments.

### Detection of cell viability

Cell viability was determined using an MTT assay. The cells (1×10^5^ cells/ml) were treated with MTT at 0.5 mg/*μ*l. The resulting purple formazan crystals were dissolved in DMSO. The solutions were then loaded in a 96-well plate, and analyzed on an automated microplate spectrophotometer (VersaMax; Molecular Devices, Silicon Valley, CA, USA) at 570 nm. MTT assay was performed in triplicate in each experiment.

### Preparation of nuclear and cytosolic extracts

To analyze Nrf2, the cells (1×10^5^ cells/ml) were resuspended at 4°C in buffer A, containing 10 mM HEPES pH 7.9, 1.5 mM MgCl_2_, 10 mM KCl, 0.5 mM dithiothreitol DTT and 0.2 mM phenylmethylsulfonyl fluoride (PMSF), allowed to swell on ice for 10 min and then vortexed for 10 sec using a Mini Vortexer (Thermo Fisher Scientific, Springfield Township, NJ, USA). The samples were centrifuged at 10,000 x g for 2 min and the supernatant, containing the cytosolic fraction, was stored at −80°C. The pellet was resuspended in cold buffer B, containing 20 mM HEPES pH 7.9, 25% glycerol, 420 mM NaCl, 1.5 mM MgCl_2_, 0.2 mM EDTA, 0.5 mM DTT, 0.2 mM PMSF, 2.5 *μ*g/*μ*l leupeptin and 2.5 *μ*g/*μ*l aprotinin, and incubated on ice for 20 min for high salt extraction. The cellular debris was removed by centrifugation at 13,000 x g for 10 min at 4°C and the supernatant fraction, containing the nuclear protein extract, was stored at −80°C. The proteins were quantified using Bradford’s reagent (Sigma-Aldrich), according to the manufacturer’s instructions. Briefly, the quantification of total proteins was performed by means of a standard curve of absorbance at 595 nm obtained from solutions containing known concentrations of BSA (0, 0.005, 0.010, 0.015, 0.020 and 0.025 mg/ml), Bradford’s reagent (0.20 ml) and sufficient water to reach a final volume of 1 ml. The samples contained 20 *μ*g of the nuclear extract and 200 *μ*l Bradford’s reagent. Following agitation, the absorbance of the samples was measured at 595 nm using an automated microplate spectrophotometer (VersaMax).

### Western blot analysis

Equal quantities of nuclear and cytosolic extracts (20 *μ*g) were electroblotted onto a nitrocellulose membrane, following separation using 8–12% sodium dodecylsulfate-polyacrylamide gel electrophoresis. The blot was probed using primary antibodies against HO-1, Nrf2, Lamin B, p-p65, p65, p-eNOS, eNOS, p-IRS-1, IRS-1, and β-actin. HRP-conjugated anti-IgG antibodies were used as the secondary antibodies to detect the previously mentioned protein bands by enhanced chemiluminescence (WESTSAVE-Up; AbFrontier, Seoul, Korea).

### Gene silencing using Nrf2 siRNA

The siRNA against Nrf2 or the control siRNA were introduced into the HUVECs by reverse transfection, using Lipofectamine™ RNAiMAX reagents (Invitrogen Life Technologies, Carlsbad, CA, USA), according to the manufacturer’s instructions. In brief, the transfection mixture was applied to a 6-well plate immediately prior to plating cells (1×10^5^ cells/ml) in complete medium (RPMI-1640 containing 10% FBS) without antibiotics. The medium was replaced with fresh medium after 4 h and the cells were incubated overnight at 37°C.

### Measurement of the production of TNF-α and IL-6

The cells, grown to confluence in 24-well plates, were pre-incubated at 37°C for 12 h with different concentrations of Pic, and then stimulated with PA (100 *μ*M) for 12 h in serum-free medium. Following collection of the medium from each well, the levels of TNF-α and IL-6 in the supernatant were assayed using human TNF-α and IL-6 enzyme-linked immunosorbent assay (ELISA) kits (R&D Systems, Minneapolis, MN, USA), according to the manufacturer’s instructions.

### Measurement of the activity of heme oxygenase

The activity of heme oxygenase was determined at the end of each treatment, as described previously (16). Briefly, microsomes from the harvested cells were (1×10^5^ cells/ml) added to a reaction mixture containing nicotinamide adenine dinucleotide phosphate (1 mM), glucose-6-phosphate dehydrogenase (10 *μ*g/ml), rat liver cytosol (20 *μ*g/ml), as a source of biliverdin reductase, and hemin (10 *μ*M). The reaction mixture was incubated in the dark at 37°C for 1 h and terminated by the addition of 1 *μ*l chloroform. Following vigorous vortexing and centrifugation at 10,000 x g for 30 min at 4°C, the quantity of extracted bilirubin in the chloroform layer was determined by measuring the difference in absorbance between 464 and 530 nm using an automated microplate spectrophotometer (VersaMax), with an extinction coefficient of 40 mmol/l^−1^·cm^−1^.

### Measurement of the intracellular formation of ROS

The intracellular formation of ROS was measured using 2′,7′-dichlorofluorescein diacetate (DCF-DA; Molecular Probes, Eugene, OR, USA) (7). This nonpolar compound is converted to the membrane-impermeant polar derivative, DCF, by esterases following uptake by the cell. DCF is nonfluorescent, however it is rapidly oxidized to the highly fluorescent DCF by intracellular ROS. In brief, the cells were seeded in 96-well black plates at a concentration of 1×10^5^ cells/*μ*l and were pre-incubated for 12 h with 20 *μ*M Pic. Following the addition of DCF-DA (10 *μ*M) at 37°C for 10 min, the cells were rinsed three times with phosphate-buffered saline (PBS), and further incubated at 37°C with media containing PA (100 *μ*M) for 2 h. Following incubation, the media was discarded, and the cells were washed with PBS. The fluorescence intensity was determined using a fluorescence plate reader (FLIPR; Molecular Devices) at 488 nm for excitation and 525 nm for emission, with results presented as the percentage of the control (treated with medium alone). For microscopic analysis, the cells were cultured on coverslips in 6-well plates and treated, as described above. The cells were incubated at 37°C with DCF-DA for 10 min in the dark, and stimulated with PA for 0.5 h. The cells were then washed twice with ice-cold PBS, fixed with 2% paraformaldehyde for 3 min and washed twice again with ice-cold PBS. The coverslips were then mounted onto slides, and analysis of ROS production was performed using a fluorescent microscope (Axiovert 200;Carl Zeiss Microimaging, Thornwood, NY, USA).

### Measurement of the intracellular production of NO

The cell membrane permeable probe, 4-amino-5-methylamino-2′,7′- difluorofluorescein (DAF-FM) diacetate (Molecular Probes), was used to detect intracellular NO. Once inside the cells, DAF-FM diacetate is deacetylated by intracellular esterases, becoming DAF-FM, which can be detected by fluorescent methods (7). Briefly, the cells were seeded in 96-well black plates at a concentration of 1×10^5^ cells/*μ*l and were pre-incubated for 12 h with different concentrations of Pic. The cells were stimulated without or with PA (100 *μ*M) for 12 h and then washed three times with PBS. Following the addition of DAF-FM diacetate (5 *μ*M) at 37°C for 0.5 h, the cells were rinsed three times with PBS and further incubated with media containing insulin (100 nM; R&D Systems) at 37°C for 2 h. Following incubation, the media was discarded and the cells were washed with PBS. The fluorescence intensity was determined using a fluorescence plate reader (Molecular Devices) at 495 nm for excitation and 515 nm for emission, with results presented as the percentage of the control. For microscopic analysis, the cells were cultured at 37°C on coverslips in 6-well plates and treated, as described above. The cells were incubated with DAF-FM diacetate for 0.5 h in the dark, and stimulated with insulin for 0.5 h. The cells were then washed twice with ice-cold PBS, fixed with 2% paraformaldehyde for 3 min and washed twice again with ice-cold PBS. The coverslips were then mounted onto slides, and analysis of the production of NO was performed using a fluorescent microscope (Carl Zeiss Microimaging).

### Measurement of NF-κB p65 DNA-binding activity

The content of NF-κB binding to DNA in nuclear extracts was measured using a specific TransAM^®^ NF-κB p65 assay kit (Active motif, Carlsbad, CA, USA), according to the manufacturer’s instructions. Briefly, a 96-well plate was precoated with an oligonucleotide, containing the NF-κB p65 binding consensus site. The active form of the p65 subunit was detected using p65 antibodies, incubated for 1 h, specific for an epitope, which was accessible only when the appropriate subunit bound to its target DNA. An HRP-conjugated secondary antibody provided a colorimetric readout, which was quantified using a spectrophotometer (450 nm).

### Measurement of Nrf2 DNA-binding activity

The DNA binding of Nrf2 was measured using a specific TransAM^®^ Nrf2 assay kit (Active Motif), according to the manufacturer’s instructions. Briefly, the nuclear extracts were incubated in the oligonucleotide-coated wells. Subsequently, the wells were washed twice with PBS and incubated with antibody against Nrf2 at 37°C. The addition of secondary antibody conjugated to HRP provided a colorimetric readout. The absorbance of each well was measured using a microplate reader at 450 nm (Molecular Devices).

### Measurement of glucose uptake

The *D*-glucose analogue, 2-Deoxyglucose (2-DG), is transported into cells and phosphorylated by the same mechanisms as glucose. Thus, 2-DG uptake is defined as glucose transport and intracellular phosphorylation by hexokinase. Phosphorylation serves to trap 2-DG inside the cells as 2-deoxyglucose 6-phosphate (2-DG6P), making it possible to determine the rates of glucose uptake over extended periods of time (17). The insulin-stimulated glucose uptake of the HUVECs was determined by measuring the transport of 2-DG into the cells. Briefly, media were removed and the cells were washed twice with Krebs-Ringer phosphate-HEPES (KRPH) buffer, containing 2% BSA followed by stimulation with or without 100 nM insulin for 0.5 h at 37°C. The cells were further incubated at 37°C for 1 h KRPH buffer, containing 2% BSA and 1 mM 2-DG. The culture plates were then placed on ice, and the cells were washed three times with PBS. The cells in six wells were collected in 500 *μ*l 10 mM Tris-HCl (pH 8.1) containing 1% Triton X-100, heated at 95°C for 15 min and centrifuged at 17,800 x g for 15 min at 4°C. A portion of the resulting supernatant was diluted 20 times with 10 *μ*M Tris-HCl (pH 8.1) and the 2-DG6P content was analyzed. The 2-DG6P content was determined by measuring the quantity of NADPH produced during 2-DG6P oxidation according to the manufacturer’s instructions using the appropriate commercially available ELISA kit (Abcam, Cambridge, MA, USA).

### Statistical analysis

The results of all the experiments are expressed as the mean ± standard deviation of multiple experiments (n≥3). All statistical analyses were performed using SPSS version 10.0 software (SPSS Inc., Chicago, IL, USA). The data were compared using one-way analysis of variance (ANOVA) with post-hoc Bonferroni’s analysis, when applicable. P<0.05 was considered to indicate a statistically significant difference.

## Results

### Pic induces the endothelial expression of HO-1 via the activation of Nrf2

**Figure 1:**
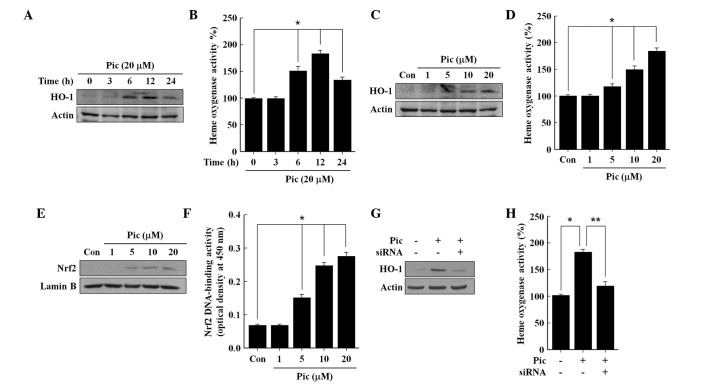
Effects of Pic on the expression of HO-1, activity of HO, nuclear accumulation of Nrf2 and Nrf2 DNA-binding activity in human umbilical vein endothelial cells. The cells were incubated with (A and B) 20 *μ*M Pic or (C and D) different concentrations of Pic for (A and B) different durations or for (C and D) 12 h. The cells were incubated for 2 h with (E and F) different concentrations of Pic, and (G and H) cells transiently transfected with either control siRNA or Nrf2 siRNA were incubated with 20 *μ*M of Pic for 12 h. Western blot analysis for the (A, C and G) expression of HO-1 and (E) nuclear accumulation of Nrf2 nuclear was performed. Untreated cells served as controls (Con). Representative blots, selected from three separate experiments are shown. The (B, D and H) activity of HO and DNA-binding activity of (F) Nrf2 were measured. All data are expressed as the mean ± standard deviation of three independent observations in separate cell culture wells. ^*^P<0.01 and ^**^P<0.05. HO, heme oxygenase; Pic, piceatannol; siRNA, small interfering RNA; Nrf2, nuclear factor erthyroid-2-related factor-2.

The HUVECs were treated with different concentrations of Pic, and an MTT assay for cell viability was performed after 24 h incubation. A survival rate of ~95% was observed at a Pic concentration of 20 *μ*M, however, significant cytotoxic signs were observed >25 *μ*M Pic (data not shown). Thus, for the subsequent experiments, the maximum concentration of Pic was limited to 20 *μ*M. Upon exposure to the non-cytotoxic 20 *μ*M of Pic, the HO-1 enzyme was expressed after 6 h and increased until 12 h, following which it reduced until 24 h (Fig. 1A), which corresponded to the levels of HO activity (Fig. 1B). Pic increased the expression of HO-1 (Fig. 1C) and the activity of HO (Fig. 1D) in a concentration-dependent manner, confirming that the upregulation of HO-1 was accompanied by increased HO activity in the Pic-treated cells. In addition, Pic increased the content of Nrf2 in the nuclear fraction, in a concentration-dependent manner (Fig. 1E), and consequently enhanced the DNA-binding activity of Nrf2 (Fig. 1F). To confirm the role of Nrf2 in Pic-induced expression of HO-1, the present study subsequently assessed the effect of transient transfection with Nrf2 siRNA on the Pic-induced expression of HO-1. Silencing with Nrf2 siRNA reduced the protein levels of Nrf2 compared with the negative controls in the total cell lysates (data not shown). The Pic-induced expression of HO-1 (Fig. 1G) and activity of HO (Fig. 1H) were eradicated by transfection with Nrf2 siRNA, suggesting that Pic-induced expression of HO-1 requires the activation of Nrf2.

### Pic reduces the PA-induced inflammatory response and formation of ROS

**Figure 2:**
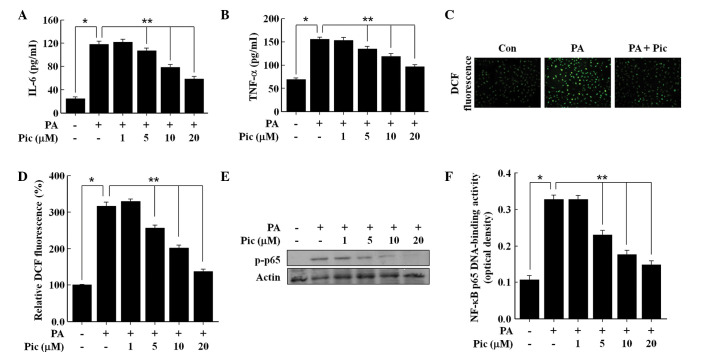
Effects of Pic on the secretions of IL-6 and TNF-α, formation of ROS, phosphorylation of p65, and DNA-binding activity of NF-κB in human umbilical vein endothelial cells stimulated with PA. The cells were pretreated for 12 h with different concentrations of Pic or with 20 *μ*M of Pic, and then exposed to 100 *μ*M PA for (A and B) 12 h, (C) 0.5 h or (D, E and F) 2 h. Untreated cells served as controls (Con). For analyses, (A and B) enzyme-linked immunosorbent assays were performed for cytokine secretion, fluorescence microscopic analysis was performed for the (C) formation of ROS, (D) DCF fluorescence intensity was performed for the formation of ROS, (E) western blot analysis was performed for (E) NF-κB subunit p65 phosphorylation and (F) NF-κB p65 DNA-binding activity were measured. (C) Representative fluorescent images demonstrate the increase in green fluorescence intensity of DCF produced by ROS (magnification, ×400). Representative blots or pictures, selected from three separate experiments are shown. All data are expressed as the mean ± standard deviation of three independent observations in separate cell culture wells. ^*^P<0.01 and ^**^P<0.05. Pic, piceatannol; IL, interleukin; TNF, tumor necrosis factor; ROS, reactive oxygen species; NF-κB, nuclear factor-κB; p-phosphorylated; PA, palmitic acid; DCF, 2′,7′-dichlorofluorescein diacetate.

As shown in Fig. 2, exposure of the HUVECs to the free fatty acid PA (100 *μ*M) markedly increased the secretions of TNF-α and IL-6, the production of ROS, and the activation of NF-κB, which was consistent with previously published reports (7,18). Pretreatment with Pic for 12 h effectively inhibited the PA-induced secretions of TNF-α and IL-6 in a concentration dependent manner (Fig. 2A and B), and also reduced the production of ROS (Fig. 2C and D). Similarly, Pic decreased the PA-induced phosphorylation of NF-κB p65 (Fig. 2E), a marker of NF-κB activation, and therefore, reduced PA-induced NF-κB transcriptional activity (Fig. 2F). These data demonstrated the anti-inflammatory and antioxidant actions of Pic in endothelial cells exposed to PA.

### Anti-inflammatory and antioxidant effects of Pic are mediated via the activation of HO-1

**Figure 3:**
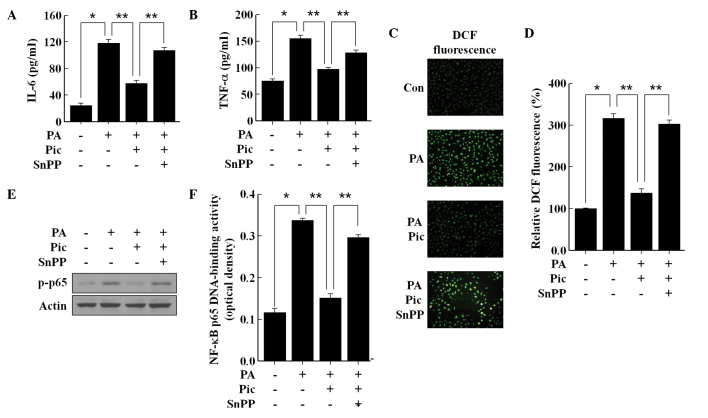
Involvement of the expression of HO-1 in the anti-inflammatory and anti-oxidative effects of Pic in human umbilical vein endothelial cells stimulated with PA. The cells were pretreated for 12 h with 20 *μ*M of Pic and then exposed to 100 *μ*M of PA for (A and B),12 h (C) 0.5 h or (D, E and F) 2 h in the presence or absence of 20 *μ*M SnPP. Untreated cells served as controls (Con). For analyses, (A and B) enzyme-linked immunosorbent assays were performed for cytokine secretion, (C) fluorescence microscopic analysis was performed for the formation of RO, (D) DCF fluorescence intensity was performed for ROS formation (E) Western blot analysis was performed for the phosphorylation of NF-κB subunit p65 and DCF fluorescence intensity was performed for NF-κB p65 DNA-binding activity. (C) Representative fluorescent images demonstrate the increase in green fluorescence intensity of DCF produced by ROS (magnification, ×400). Representative blots or pictures, selected from three separate experiments are shown. All data are expressed as the mean ± standard deviation of three independent observations in separate cell culture wells. ^*^P<0.01 and ^**^P<0.05. Pic, piceatannol; PA, palmitic acid; snPP, tin protoporphryin-IX; IL, interleukin; TNF, tumor necrosis factor; ROS, reactive oxygen species; NF-κB, nuclear factor-κB; p-phosphorylated; DCF, 2′,7′-dichlorofluorescein diacetate.

As HO-1 is understood to suppress inflammation and the formation of ROS in endothelial cells (8,9), the present study investigated whether the observed anti-inflammatory and antioxidant actions of Pic can be mediated via HO-1 enzymatic activation. SnPP was used to suppress HO-1 enzymatic activity, and the results revealed that the inhibition of IL-6 (Fig. 3A) and TNF-α (Fig. 3B) production by Pic pretreatment was significantly reversed by SnPP in the PA-stimulated endothelial cells. SnPP also eradicated the antioxidant effect of Pic. Following inhibition of the activity of HO-1 by SnPP, Pic pretreatment failed to prevent PA-induced ROS formation (Fig. 3C and D). Notably, SnPP significantly impaired the inhibitory effect of Pic on the phosphorylation of NF-κB p65 (Fig. 3E) and transcriptional activity of NF-κB (Fig. 3F) in the PA-stimulated endothelial cells. These data demonstrated that HO-1 enzymatic activity was essential for the anti-inflammatory and antioxidant actions of Pic in the PA-stimulated HUVECs.

### Pic reduces PA-induced insulin resistance and eNOS dysfunction via the activation of HO-1

**Figure 4:**
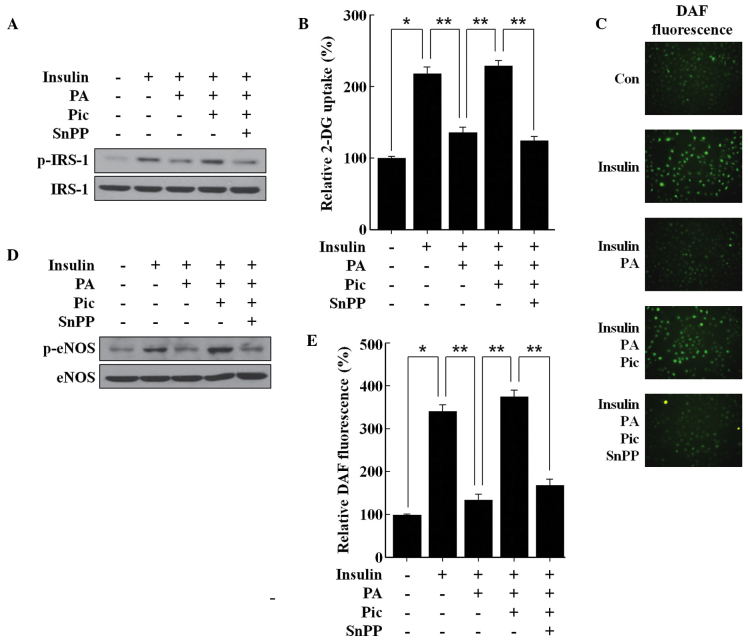
Effects of Pic on insulin-mediated IRS-1 tyrosine phosphorylation, glucose uptake, activation of eNOS, and productionof NO in human umbilical vein endothelial cells stimulated with PA. The cells were pretreated for 12 h with 20 *μ*M Pic and then exposed to 100 *μ*M PA for 12 h in the presence or absence of 20 *μ*M of SnPP. The cells were stimulated with 100 nM insulin for (A, B, C and D) 0.5 h or (E) 2 h. (A) Western blot analysis was performed for IRS-1 tyrosine phosphorylation and (D) the activation of eNOS, (C) fluorescence microscopic analysis was performed for the production of NO. (B) Glucose uptake and the (E) DAF-FM fluorescence intensity of the production of NO were also determined. (C) Representative fluorescent images demonstrate the increase in green fluorescence intensity of DAF produced by NO (magnification, ×400). Representative blots or pictures, selected from three seperate experiments are shown. All data are expressed as the mean ± standard deviation of three independent observations in separate cell culture wells. ^*^P<0.01 and ^**^P<0.05. Pic, piceatannol; PA, palmitic acid; snPP, tin protoporphryin-IX; NO, nitric oxide; eNOS, endothelial NO synthase; p-phosphorylated; DAF,-FM, 4-amino-5-methylamino-2′,7′-difluorofluorescein.

In the HUVECs, PA attenuated the insulin-mediated tyrosine phosphorylation of IRS-1 and consequently reduced glucose uptake (Fig. 4A and B), suggesting that PA may induce insulin resistance. PA also reduced the insulin-mediated phosphorylation of eNOS and the subsequent production of NO (Fig. 4C–E), suggesting that PA may induce eNOS dysfunction. Pic effectively prevented the inhibitory effect of PA on the insulin-mediated tyrosine phosphorylation of IRS-1 (Fig. 4A) and uptake of glucose (Fig. 4B). Pic also restored the loss of insulin-mediated NO production (Fig. 4C and F) by mitigating the inhibitory effect of PA on the insulin-mediated phosphorylation of eNOS (Fig. 4D). Notably, these beneficial effects of Pic against PA insult were significantly reversed following inhibition of HO-1 activity by SnPP (Fig. 4), demonstrating that HO-1 enzymatic activity was essential, at least in part, for the observed protective effects of Pic, in endothelial cells exposed to a high concentration of PA.

## Discussion

PA is a circulating free fatty acid, which is often observed at a high concentration in insulin-resistant states (19) and has been observed to induce inflammation and the formation of ROS, and decrease insulin-mediated eNOS activity, which are the causes of endothelial dysfunction, in an endothelial cell culture model (7). The present study demonstrated that, in HUVECs stimulated with PA, pretreatment with the resveratrol analogue, Pic, prevented inflammation and the formation of ROS, and also attenuated the reduction of insulin-mediated eNOS activity and production of NO. In addition, the observed protective effects of Pic were associated with its induction of the expression of HO-1, which is well-known to preserve endothelial function (8).

Several, but not all, naturally occurring compounds with anti-inflammatory and antioxidant activities are known to induce the expression of HO-1 and to exert their beneficial effects through the HO-1-dependent pathway (10). It has been reported previously that Pic, a naturally occurring hydroxylated analog of resveratrol, is capable of inducing the expression of HO-1 through the activation of Nrf2 in neuronal cells (20) and epithelial cells (21). Notably, Pic is a more potent inducer of HO-1 inducer compared with resveratrol in macrophages (22), suggesting that the existence of the additional hydroxyl group in Pic may be critical in its induction of the expression of HO-1. A previous study demonstrated that Pic induces the expression of HO-1 expression in HUVECs (23). The present study further investigated the mechanism underlying the altered expression of HO-1 following the treatment of HUVECs with Pic, and revealed that the effect was dependent on the activation of Nrf2. In addition to the anti-inflammatory and antioxidant properties of endothelial HO-1 *in vitro* (9), it is also beneficial *in vivo* in animal models of atherosclerosis and restenosis (9). In this respect, the present study aimed to examine the potential positive effect of the expression of HO-1 by Pic on endothelial dysfunction in HUVECs, an endothelial cell culture model.

The elevation of circulating FFAs is considered to be associated with to the onset and progression of endothelial dysfunction and associated diseases (2). It has been noted that FFAs may increase the production of pro-inflammatory cytokines and generation of ROS via the activation of NF-κB in human endothelial cells (3). Pro-inflammatory cytokines and ROS have been observed to impair eNOS function and reduce the believability of NO, possibly by disrupting the action of insulin (19). PA, a circulating FFA, acts as a natural dietary ligand for the activation of TLR4 signal transduction, which activates NF-κB in various types of cell, including endothelial cells (3), promotes the release of pro-inflammatory cytokines, including TNF-α and IL-6, and promotes the formation of ROS (7). The results of the present study demonstrated that PA induced the phosphorylation of NF-κB p65 and increased the DNA-binding activity of NF-κB p65, resulting in increased production of TNF-α and IL-6. PA also induced intracellular ROS formation. Notably, pretreatment with Pic suppressed the PA-induced activation of NF-κB and formation of ROS, and decreased the production of TNF-α and IL-6. PA attenuated IRS-1 tyrosine phosphorylation and glucose uptake in response to insulin, leading to impairment of downstream insulin signaling, evidenced by reduced the phosphorylation of eNOS and production of NO. However, these effects of PA were effectively reversed by Pic pretreatment. Given the involvement of inflammatory and oxidative stresses in endothelial dysfunction (8,9), suppression of the NF-κB-dependent inflammatory response and production of ROS by Pic may have be responsible for its restoration of the loss of insulin-mediated phosphorylation of eNOS and production of NO. However, the precise mechanism underlying the anti-inflammatory and antioxidant properties of Pic remain to be fully elucidated. Previous studies have demonstrated that Pic induces the expression of HO-1, which can exert anti-inflammatory and antioxidant effects (20,22) and the present study revealed that Pic increased the expression of HO-1 via the Nrf2 pathway in HUVECs. Therefore, the present study investigated whether the expression of HO-1 contributed the to anti-inflammatory and antioxidant effects of Pic, at least, under the experimental conditions assessed. The inhibition of HO-1 activity by SnPP eradicated the anti-inflammatory and antioxidant effects of Pic, and reversed the restored insulin-mediated phosphorylation of eNOS and production of NO, suggesting that the anti-inflammatory and antioxidant effects of Pic against PA insult may be associated, at least in part, with the expression of HO-1.

In conclusion, the present study demonstrated that the pretreatment of HUVECs with Pic resulted in Nrf2-dependent expression of HO-1. Furthermore, the expression of HO-1 by Pic inhibited the PA-induced inflammatory response andformation of ROS, and attenuated the PA-induced reduction in the activation of eNOS and production of NO. These results indicated that Pic was protective against PA-induced endothelial dysfunction by inducing the Nrf2-dependent expression of HO-1, suggesting a potential strategy of targeting the expression of HO-1 by Pic for endothelial protection in the presence of high levels of PA, including that in obesity, diabetes and other metabolic inflammatory diseases. However, further investigation is required on the bioavailability and toxicity of Pic in humans.

## Abbreviations

2-DG: 2-deoxyglucose
eNOS: endothelial nitric oxide synthase
FFA: free fatty acid
HO-1: heme oxygenase-1
NO: nitric oxide
DAF-FM: 4-amino-5-methylamino-2′,7′-difluoro fluorescein
DCF-DA: 2′,7′-dichlorofluorescein diacetate
DAF-FM: 4-amino-5-methylamino-2′
HUVEC: human umbilical vein endothelial cell
IL-6: interleukin-6
IRS-1: insulin receptor substrate-1
NF-κB: nuclear factor-κB
Nrf2: nuclear factor E2-related factor 2
PA: palmitic acid
Pic: piceatannol
ROS: reactive oxygen species
siRNA: small interfering RNA
SnPP: tin protoporphryin-IX
TLR4: toll-like receptor 4
TNF-α: tumor necrosis factor-α
ELISA: enzyme-linked immunosorbent assay
